# Relationship of weight-adjusted waist index and developmental disabilities in children 6 to 17 years of age: a cross-sectional study

**DOI:** 10.3389/fendo.2024.1406996

**Published:** 2024-07-04

**Authors:** Xueying Li, Qi Zhou

**Affiliations:** Department of Neonatal, Children's Medical Center, The First Hospital of Jilin University, Changchun, China

**Keywords:** developmental disabilities, weight-adjusted waist index, obesity, abdominal fat, National Health and Nutrition Examination Survey

## Abstract

**Purpose:**

The development of multiple system diseases is increased by obesity. However, the connection between obesity and developmental disabilities (DDs) in children is unclear. As an obesity index, the weight-adjusted waist index (WWI) assessed fat distribution and muscle mass. In this study, we examined the correlation between WWI and DDs among children 6 to 17 years of age.

**Methods:**

This study used data from the National Health and Nutrition Examination Survey database (NHANES) covering 2003 to 2018, which included the data of 17,899 participants between 6 and 17 years of age. Data regarding their waist circumference, weight, and DDs were collected via physical examinations and questionnaire, respectively. A person’s WWI is calculated by dividing their waist circumference by their weight squared. The correlation between WWI and DDs was studied using weighted multiple logistic regression models. Additionally, a sensitivity analysis was conducted utilizing a generalized additive model and smooth curve fitting.

**Results:**

After adjusting for all covariates, WWI was positively related to DDs in children ages 6-17. Based on the sensitivity analysis, the correlation between the WWI and prevalence of DDs remained consistent across subgroups. Additionally, there was a J-shaped correlation between the WWI and the prevalence of DDs in children ages 6 through 11.

**Conclusion:**

Children 6–17 years of age with a high WWI were at greater risk for DDs; however, the causal relationships and potential mechanisms require further exploration.

## 1 Introduction

Developmental disabilities (DDs) are lifelong conditions characterized by dysfunctions in growth and development, learning and language abilities, and behavioral development (1). Common DDs include attention deficit hyperactivity disorder (ADHD), autism spectrum disorder (ASD), intellectual disabilities (ID), learning disabilities, epilepsy, and cerebral palsy (2). An overall increase in DDs among children has been observed. Among children 3-17 years of age in the United States, the prevalence of DDs increased from 12.84% to 15.04% between 1997 and 2008, and from 16.2% to 17.8% between 2009 and 2017 (3). Additionally, from 2003 to 2017, the incidence rate (per 10,000 individuals) of DDs in South Korea doubled, and their prevalence rate (per 100 individuals) increased four-fold (4). Taiwan’s incidence of DDs among children younger than 6 years of age also increased by 20% between 1997 and 2008 (5). These changes may be attributable to increased awareness, recognition, and access to healthcare services (1). Typically, children with DDs require special education to address their behavioral and developmental challenges. Children with DDs face greater risks associated with their health, academic performance, and well-being than children without DDs. As a result, caregivers of children with DDs experience a greater level of stress (6).

Obesity is a global problem (7). There has been an increase in the incidence of obesity among children and adolescents due to contemporary eating habits and lifestyles (8). During the period 1975-2016, obesity prevalence in children and teenagers has gradually increased globally (9). It is estimated that 50 million girls and 74 million boys worldwide are obese in 2016. Obesity is associated with many physical and psychological illnesses, including hypertension (10), cardiovascular disease (11), depression (12), and all-cause mortality (13). Obesity in childhood may persist into adolescence and adulthood, and it is highly associated with cardiometabolic diseases, cancer, and premature mortality in adulthood (11). Body mass index (BMI) and waist circumference (WC) are commonly used to measure obesity. However, individuals diagnosed with overweight or obesity using the BMI and WC may have a lower risk for disease-related mortality than healthy-weight individuals. Additionally, some overweight or obesity cases have beneficial effects on patients; this is known as the “obesity paradox” (14). Consequently, whether the BMI and WC can accurately and effectively assess obesity has been questioned. Therefore, the identification of a metric that eliminates the obesity paradox was important to the accuracy of this study. Weight-adjusted waist index (WWI) was first reported in 2018 by park et al. (15). WWI is calculated as the ratio between the WC (cm) and the square root of the weight (kg), which serves as a index to assess fat distribution and muscle mass and can reflect non-weight-dependent central obesity (15). Currently, the WWI is associated with depression, cognitive impairment, and bone mineral density (16–18). However, there have been no studies of childhood DDs related to the WWI. It has been demonstrated in previous studies that DDs are associated with childhood overweight and obesity (19–21). However, the correlation of the WWI with the prevalence of DDs has not been studied. Therefore, it is unclear whether obesity is associated with DDs, and more developmental indicators are required to assess the relationships between growth, development, and DDs among children. An objective of this study was to determine if there was a correlation between WWI and DDs in children aged 6-17 years old.

## 2 Methods

### 2.1 Data acquisition and ethics statement

This data was obtained from the National Health and Nutrition Examination Survey (NHANES), an annual national survey conducted by the Centers for Disease Control and Prevention as well as the National Center for Health Statistics. The NHANES investigated the health status and nutritional status of adults and children in the United States through a series of cross-sectional, complex, and multistage surveys conducted over the course of more than 50 years. The NHANES combined questionnaires, physical examinations, and laboratory examinations to collect information based on a nationally representative sample in the United States (22). A study protocol for NHANES was approved by the Research Ethics Review Board of the Center for Health Statistics, and written informed consent was obtained from all participants or their guardians. Subsequently, the NHANES data were made available to the public after anonymization. NHANES’ website provides more information about this study (http://www.cdc.gov/nchs/nhanes).

### 2.2 Participant selection

**Figure 1:**
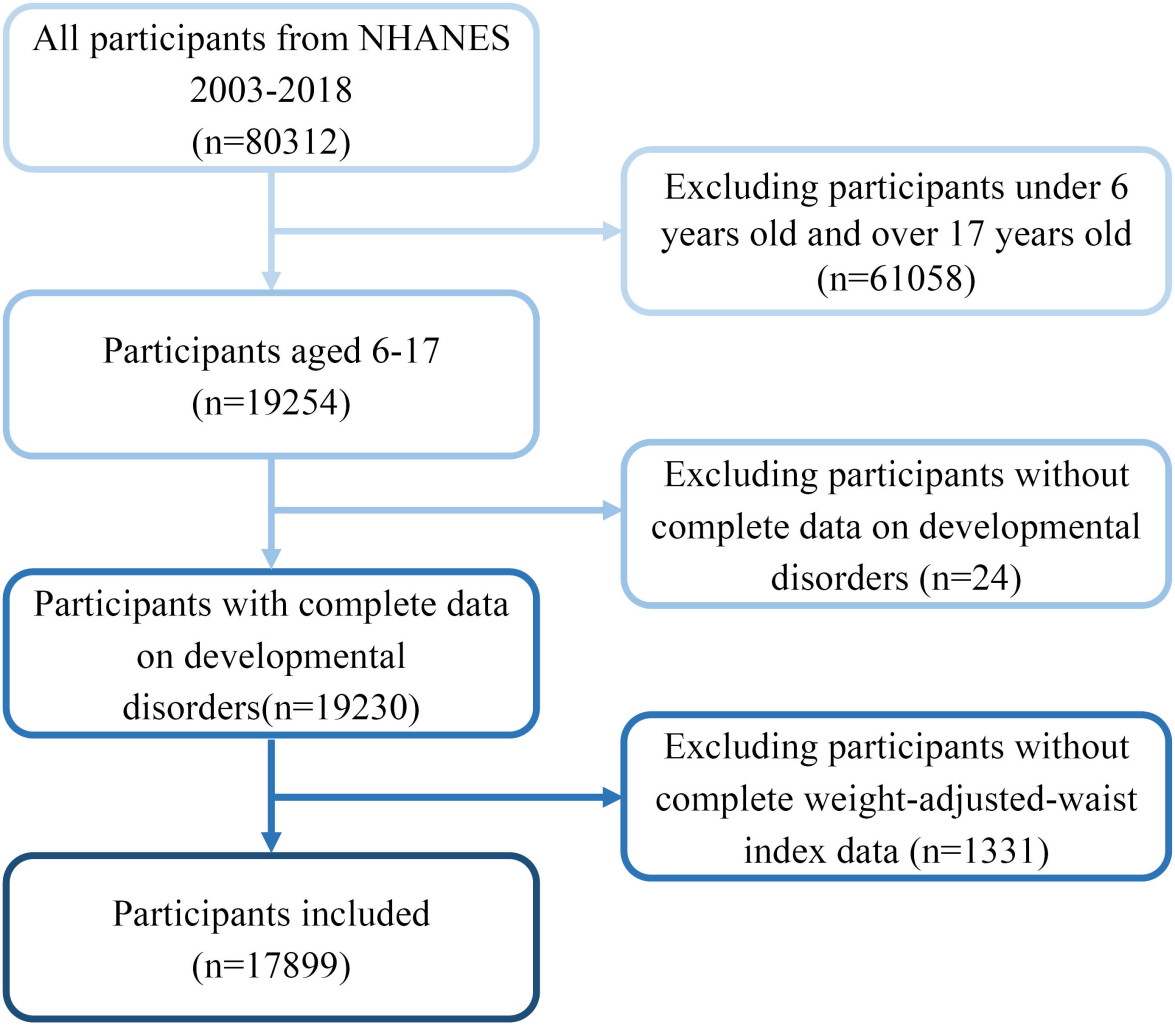
The data screening process for this study.

A total of 80,312 NHANES data points were obtained between 2003 and 2018. However, only 19,254 data points were eligible to be included in the final analysis after limiting the participants’ ages to 6-17. Subsequently, 24 and 1331 participants with missing WWI and DD data, respectively, were excluded, and 17,899 participants were included in the study (**Figure 1**).

### 2.3 Variables included in the present work

During this study, parents of participants were asked the following question: “Does your child receive special education or early intervention services?”. If the answer was yes, then the participant was diagnosed with DDs. Special education and early intervention for children with DDs improve their quality of life and skills development (23, 24). The WC (cm) and body weight (kg) were derived from the physical examination data. WWI was determined by dividing the WC by the square root of the weight of each participant, with two decimal places being maintained. WWI was used as a continuous variable in this study and grouped into tertiles for further analysis.

### 2.4 Assessment of covariates

During this study, various variables, including age, sex, ethnicity, educational background, poverty-to-income ratio (PIR), birth weight, and serum cotinine levels, were considered (17, 25, 26). PIR was classified according to household income and poverty as follows: low income (PIR ≤1.3); medium income (PIR 1.3–3.5); and high income (PIR ≥3.5). Furthermore, education levels were categorized as follows: less than ninth grade; 9th to 11th grade; high school; and college or associate’s degree. Children with a birth weight less than 5 pounds were defined as the low-birth-weight group. Serum cotinine levels served as a proxy for tobacco exposure; a cutoff value of 0.05 ng/mL was used to differentiate between individuals with and without exposure. There were also three subgroups of serum cotinine levels: no exposure to tobacco (cotinine level 0.05 ng/mL), passive exposure to tobacco (cotinine level 0.05-2.99 ng/mL), and active exposure to tobacco (2 ng/mL).

### 2.5 Statistical analysis

A combination of R software version 4.3.0 and EmpowerStats version 4.1 was used in the analysis of the data, and P < 0.05 was used as the threshold for statistical significance. For continuous and categorical variables, weighted t and chi-square tests were conducted, and the results were provided as weighted mean ± SE and weighted % (95% confidence interval), respectively. The correlation between the WWI and DDs was analyzed using a weighted multiple logistic regression analysis. Model 1 did not include adjusted variables. Model 2 was adjusted for age, sex, race, PIR, and education level. In model 3, all covariates have been taken into account. Additionally, DDs and WWI were examined in subgroups based on age, sex, PIR, serum cotinine levels, and birth weight. Furthermore, smooth curve fitting approach and a generalized additive model were used to determine the linear correlation between WWI and DDs after excluding all covariates. Simultaneously, a piecewise regression model fitting analysis was performed to determine the threshold effect, and logarithmic similarity random ratio tests were performed to evaluate the existence of a threshold. Finally, by using a two-step recursive method, the inflection point (k) was identified.

## 3 Results

### 3.1 Baseline data of participants

**Figure 2:**
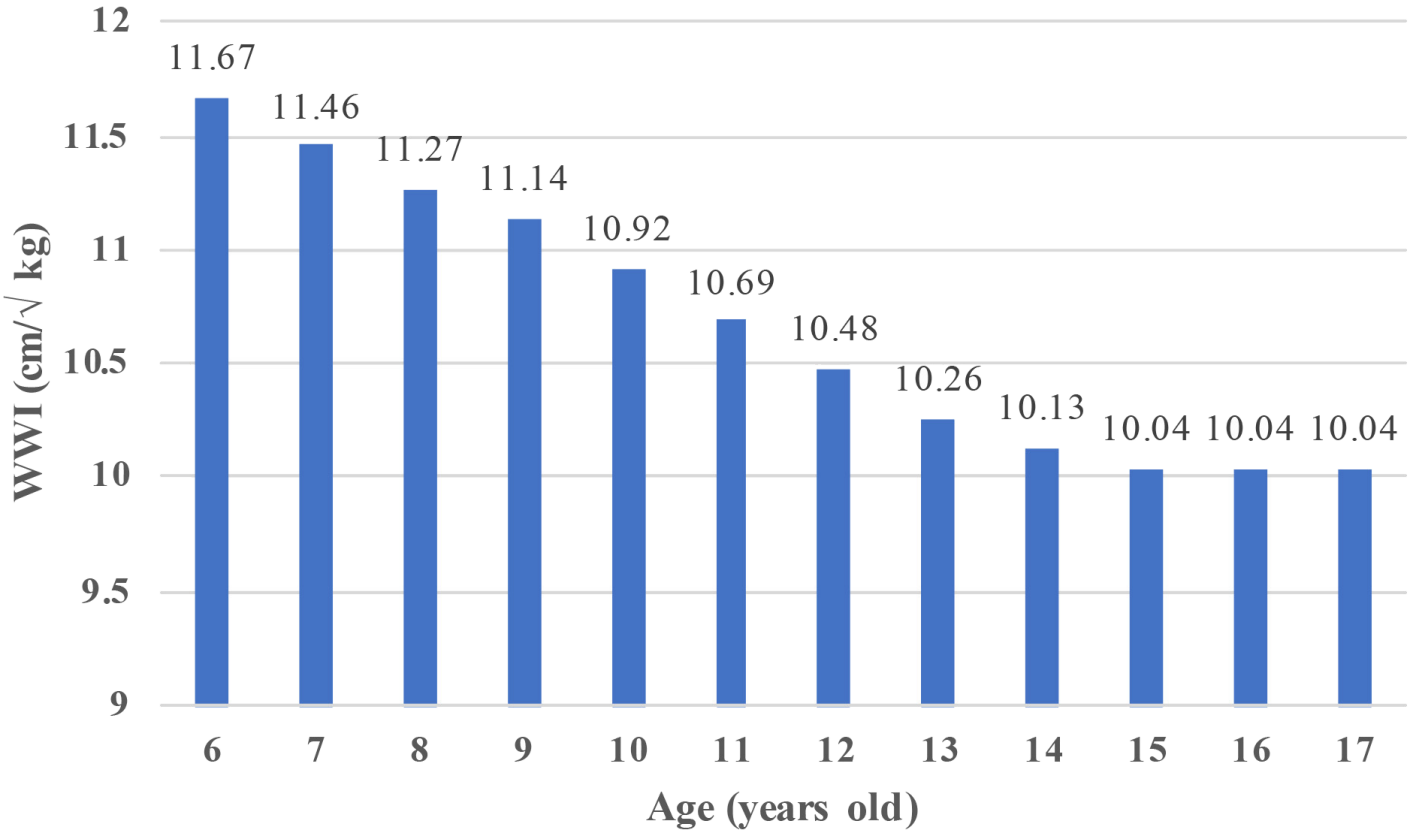
WWI trends in children aged 6–17 years WWI, weight-adjusted waist index.

This study included 17,899 children ages 6–17. A description of the baseline characteristics of the participants was presented in **Table 1**. In terms of gender, 50.8% of the participants (n=9092) were males, and 49.2% were females (n=8807). Regarding race, 27.53% and 27.47% of the participants were non-Hispanic white and non-Hispanic black, respectively, and 25.46% were Mexican American. Moreover, 10.69% and 8.87% of the participants were other race or multi-racial and other Hispanic race, respectively. Additionally, 91.47% (n=13,635) of children had a low birth weight. The mean total WWI of the population was 10.69 ( ± 0.92). Children with and without DDs had significant differences in age, sex, race, PIR, education level, birth weight, tobacco exposure, and WWI (p<0.05). Children with DDs had a higher percentage of males, low family incomes, low education levels, low birth weights, passive tobacco exposure, and lower WWI rates than children without DDs. Additionally, we calculated the average WWI of children of different ages (**Figure 2**). The results showed that between the ages of 6 and 14 years, the WWI gradually decreased with increasing age, whereas the WWI remained stable between the ages of 15 and 17 years.

**Table 1:** Baseline data of participants.

Variables	All (n=17899)	Non-DDs (n=16111)	DDs (n=1788)	P-value*
**Age (year)**	11.59 ± 0.08	11.67 ± 0.08	11.17 ± 0.22	0.0002
**BMI (kg/m^2^)**	20.98 ± 0.13	20.97 ± 0.14	21.10 ± 0.37	0.4903
**WWI (cm/√ kg)**	10.68 ± 0.02	10.66 ± 0.03	10.85 ± 0.05	<0.0001
**Sex (%)**				<0.0001
Male	51.12 (50.06, 52.17)	49.33 (48.25, 50.42)	67.15 (64.20, 69.98)	
Female	48.88 (47.83, 49.94)	50.67 (49.58, 51.75)	32.85 (30.02, 35.80)	
**Race or Ethnicity (%)**				0.0008
Mexican American	14.33 (12.47, 16.42)	14.78 (12.85, 16.94)	10.34 (8.33,12.77)	
Other Hispanic	6.77 (5.84, 7.84)	6.69 (5.76, 7.75)	7.52 (5.85, 9.62)	
Non-Hispanic White	55.97 (52.86, 59.04)	55.86 (52.71, 58.95)	57.03 (52.76, 61.19)	
Non-Hispanic Black	14.31 (12.72, 16.05)	14.05 (12.48, 15.79)	16.56 (14.28, 19.13)	
Other Race/Multi-Racial	8.61 (7.69, 9.63)	8.62 (7.70, 9.64)	8.55 (6.57, 11.05)	
**Family PIR (%)**				<0.0001
Low PIR	30.66 (28.72, 32.68)	29.52 (27.59, 31.53)	40.72 (36.90, 44.65)	
Middle PIR	37.85 (36.28, 39.44)	37.75 (36.06, 39.48)	38.70 (35.36, 42.15)	
High PIR	31.49 (29.25, 33.81)	32.72 (30.41, 35.13)	20.58 (17.52, 24.02)	
**Education level (%)**				<0.0001
Less than 9th grade	86.65 (84.76, 86.50)	85.10 (84.20, 85.95)	90.65 (88.61, 92.35)	
9-11th grade	13.81 (12.99, 14.68)	14.31 (13.48, 15.18)	9.34 (7.63, 11.37)	
High school graduate	0.37 (0.27, 0.51)	0.41 (0.30, 0.56)	0.00 (0.00, 0.00)	
Some college or AA degree	0.17 (0.10, 0.27)	0.18 (0.11, 0.30)	0.02 (0.00, 0.11)	
**Serum cotinine (%)**				<0.0001
Unexposed	55.86 (53.73, 57.97)	57.21 (55.07, 59.33)	43.57 (39.37, 47.87)	
Passively Exposed	37.18 (35.40, 38.99)	36.27 (34.43, 38.15)	45.47 (41.82, 49.17)	
Actively Exposed	6.96 (6.16, 7.85)	6.52 (5.71, 7.44)	10.96 (8.86, 13.47)	
**Low birthweight (%)**				<0.0001
No	11.68 (10.95, 12.46)	10.84 (10.09, 11.65)	18.86 (16.33, 21.69)	
Yes	88.32 (87.54, 89.05)	89.16 (88.35, 89.91)	81.14 (78.31, 83.67)	
WWI (tertiles, %)				
Low	33.55 (32.25, 34.88)	34.38 (33.00, 35.79)	26.10 (23.48, 28.92)	<0.0001
Middle	33.74 (32.76, 34.73)	33.69 (32.67, 34.72)	34.17 (31.26, 37.21)	
High	32.71 (31.52, 33.93)	31.93 (30.64, 33.25)	39.73 (36.63, 42.91)	

Values are weighted mean ± SE or weighted % (95% confidence interval).

*Calculated after weighing.

DDs, Developmental disorders; PIR, Ratio of family income to poverty; BMI, body mass index; WWI, weight-adjusted waist index, Other races include American Indian or Alaska Native, Native Hawaiian or other Pacific Islander, and multiracial persons.

### 3.2 Association between the WWI and DDs

Based on a weighted logistic regression analysis, the association between WWI and DDs was examined (**Table 2**). In model 1, which was unadjusted, the continuous variable WWI showed a positive association with DDs (odds ratio [OR], 1.26; 95% confidence interval [CI], 1.17–71.36). In model 2, with partial adjustment of covariates (OR, 1.41; 95% CI, 1.27–1.57), and model 3, with complete adjustment of covariates (OR, 1.31; 95% CI, 1.12–1.53), the WWI and DDs had a significant positive correlation. Model 3 indicates that a 1-unit increase in WWI is associated with a 31% increase in prevalence of DDs. Additionally, after converting the WWI into tertile variables, the risk of the development of DDs in children with a WWI in the third tertile was 1.55-times that of children with a WWI in the first tertile (OR, 1.55; 95% CI, 1.0–2.22). Furthermore, with each increase in the WWI, the risk of DDs for children 6–17 years of age gradually increased significantly (P for trend=0.0245).

**Table 2:** Logistic regression analysis of WWI and DDs.

Exposure	Model 1 [OR (95% CI)	Model 2 [OR (95% CI)	Model 3 [OR (95% CI)
WWI (cm/√ kg)	1.26 (1.17,1.36)	1.41 (1.27,1.57)	1.31 (1.12,1.53)
WWI (tertiles)			
T1	Reference	Reference	Reference
T2	1.34 (1.14,1.57)	1.53 (1.23,1.87)	1.44 (1.09,1.88)
T3	1.64 (1.38,1.95)	1.90 (1.46,2.46)	1.55 (1.08,2.22)
P for trend	<0.0001	<0.0001	0.0245

Model 1: no covariates were adjusted for.

Model 2: adjusted for age, sex, race, family PIR, and education level.

Model 3: all covariates presented in **Table 1** were adjusted.

WWI, weight-adjusted waist index; T, tertiles; DDs, developmental disorders.

### 3.3 Subgroup analysis

**Figure 3:**
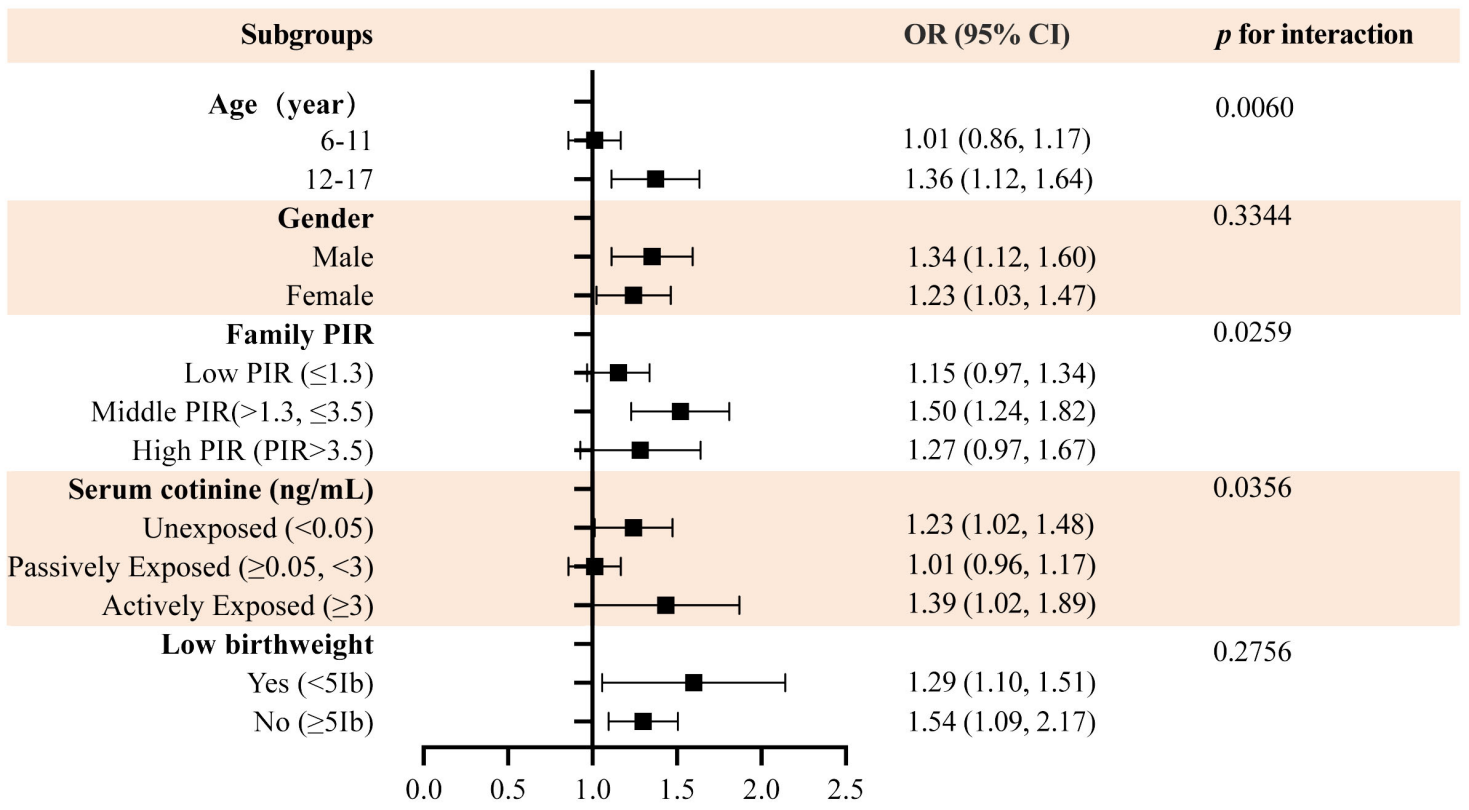
Subgroup analysis of WWI and DDs.

In order to determine whether the association between WWI and DDs was consistent among various groups, a regression analysis was conducted. In all subgroups of participants, we found a consistent positive association between WWI and DDs (**Figure 3**). Additionally, after adjusting for all covariates, age, PIR, and serum cotinine levels were effective moderators of the association between the WWI and risk of DDs; among these covariates, age 12–17 years, middle income status, no tobacco exposure, and active tobacco use significantly affected the relationship between the WWI and DDs. Finally, in terms of WWI and the prevalence of DDs, sex and low birth weight did not interact significantly.

### 3.4 Smooth curve fitting and threshold effect analysis

**Figure 4:**
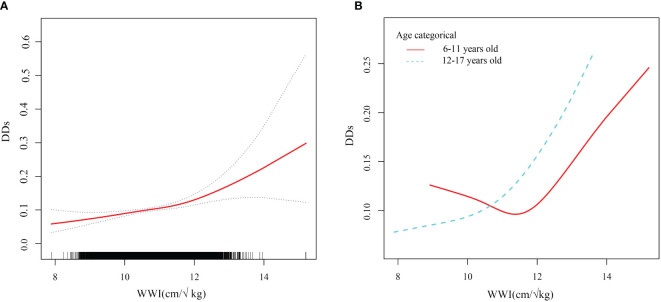
Smooth curve fitting of WWI and DDs. WWI weight-adjusted waist index; DDs developmental disabilities. **(A)** Linear relationship between WWI and DDs by the generalized additive model. **(B)** The association between WWI and DDs stratified by age.

An analysis of smooth curve fitting and a threshold effect analysis were conducted to analyze the linear relationship between WWI and DDs (**Figure 4A**; **Table 3**). As shown in **Figure 4B**, the WWI showed a J-shaped relationship with DDs among children aged 6 to 11 years (**Figure 4B**), indicating that the prevalence of DDs was the lowest when the WWI was 11.6 cm/√kg (P for non-linearity=0.0020). When WWI exceeded 11.6, DDs increased by 77% for every unit increase of WWI (OR, 1.77; 95% CI, 1.34–2.36). When the WWI was less than 11.6, the prevalence of DDs decreased with the increasing WWI; but the difference was not statistically significant (p>0.05).

**Table 3:** Threshold effect analysis of WWI and DDs.

Exposure	Model: saturation effect analysis [OR (95%CI) P value]
WWI	
Linear effect model	1.25 (1.12, 1.39) **<**0.0001
Non- linear model	
Infection point(K)	11.6
<K, effect 1	1.16 (1.02, 1.32) **0.0228**
>K, effect 2	1.56 (1.22, 2.01) **0.0005**
Log-likelihood ratio	0.055
Subgroup analysis stratified by age	
WWI turning point for 6-11years old	
Linear effect model	1.18 (1.02, 1.36) **0.029**
Non- linear model	
Infection point(K)	11.6
<K, effect 1	0.96 (0.80, 1.16) 0.693
>K, effect 2	1.77 (1.34, 2.36) **<**0.001
Log-likelihood ratio	**0.002**
WWI turning point for 12-17years old	
Linear effect model	1.34 (1.13, 1.58) **<**0.001
Non- linear model	
Infection point(K)	10.83
<K, effect 1	1.15 (0.92, 1.44) 0.230
>K, effect 2	1.84 (1.29, 2.63) **<**0.001
Log-likelihood ratio	0.051

WWI, weight-adjusted waist index.

Bold values refer to the values with statistical effects (P<0.05) in threshold effect analysis.

## 4 Discussion

DDs are lifelong diseases characterized by the impairment of one or more human body functions, including physical, learning, language, and behavioral development. Obesity is also associated with the development of DDs. However, no studies have evaluated the relationship between WWI and DDs in childhood. This study examined the association between the WWI and DDs in 17 899 children aged 6–17 years using data from the NHANES survey from 2003 to 2018. As a result of a weighted multivariable logistic regression model, it was demonstrated that a higher WWI was associated with a higher prevalence of DDs. This relationship persisted after converting the WWI into tertile variables. As demonstrated by a sensitivity analysis, the correlation between the WWI and DDs was stable and linear across groups of participants, suggesting that the WWI may be an important predictor of DDs.

A variety of diseases are associated with childhood obesity, including hypertension, insulin resistance, diabetes, and DDs (10, 27). Moreover, overweight and obese children are more likely to develop emotional and behavioral problems than children with a healthy weight (28). Halfon et al. examined 43,297 children in 10 -17 years of age and observed that overweight and obese children, as defined by the BMI, generally had a poorer health status than healthy-weight children and were more likely to develop ADHD, behavioral disorders, developmental delays, and other diseases (20). Similarly, Agranat-Meged et al. revealed that 50% of 26 hospitalized children with obesity had childhood ADHD (21). These studies suggest a close correlation between obesity and DDs.

Although there are many obesity-related indicators, the BMI is most widely used. The BMI cannot distinguish between fat weight and lean body mass and cannot assess the percentage of body fat and the distribution of obesity (29). Recently, many studies have focused on visceral fat because lipids accumulate in the skeletal muscles, liver, and pancreas (30) when the body is overloaded with nutrition, thus causing systemic metabolic disorders and increasing the risks of type 2 diabetes and heart diseases (31, 32). As WWI is derived from weight-standardized WC, it reflects central obesity, has no effect on weight, reduces the correlation between WC and BMI, and is considered more representative of abdominal fat and visceral fat (8).

According to our study, a high WWI was related to a higher prevalence of DDs. This finding is supported by several mechanisms. In the human body, fat is an endocrine organ that stores energy and plays a significant role in systemic homeostasis (33, 34). Visceral fat accumulation can interfere with endocrine metabolism because lipid metabolites are lipotoxic, hinder insulin signal transduction, and cause insulin resistance (31, 32, 35). Emotional changes associated with metabolic syndrome are primarily caused by insulin resistance (36, 37). The insulin receptor (IR) and insulin-like growth factor 1 (IGF1R) plays an important role in the development of the central nervous system (38). The common signaling cascade reactions of IR/IGF1R include PI3K/AKT/mTOR and RAS/ERK, and the activation of these pathways plays a role in neurodevelopmental disorders such as intellectual disability (ID) and ASD (38, 39). Additionally, adolescents with ASD and ADHD often have metabolic syndromes and diabetes (40). Excessive visceral fat can cause insulin resistance, leading to DDs. Researchers found that mice who were fed a western diet containing high levels of sugar and fat for three weeks developed insulin resistance, ADHD, increased impulsivity, anxious behavior, and a decrease in social interaction (41). Follow-up experiments further revealed that mice that were fed a Western diet developed movement disorders and impaired prefrontal cortex function. Reduced medial prefrontal cortex activity in humans has been reported to be a characteristic of ADHD and ASD (42, 43). Therefore, obesity-induced insulin resistance may be partially related to the risk of DDs. Second, visceral adipose tissue continues to secrete proinflammatory stimuli. Excess abdominal fat causes increased inflammation levels, a chronic low-inflammatory state, and increased expression of inflammatory factors, such as high-sensitivity C-reactive protein, leptin, IL-6, IL-1β, C-reactive protein, and tumor necrosis factor-α (44, 45). A chronic low-inflammatory state in the human body is associated with several diseases, including metabolic syndrome, insulin resistance, cardiovascular disease, and DDs. There was evidence that children with DDs, such as autism, ADHD, cerebral palsy, and epilepsy, have significantly higher levels of inflammatory factors such as IL-1, IL-6, IL-8, C-reactive protein, and tumor necrosis factor-α (46–49), than children without DDs. Additionally, a higher level of inflammatory cytokines in the body is associated with poorer behavioral scores and stereotypic behavior, and strongly correlated with the level of IL-6 in the body (50). Hughes et al. also demonstrated that CD14+ monocytes from children with ASD produce more IL-6 than those from children without DDs, and that this concentration was related to the severity of restrictive and repetitive behaviors (51). Furthermore, in terms of psychosocial health, overweight or obese children are more likely to be bullied and excluded from social settings (52). Unhealthy weight in childhood significantly impacts the quality of life and future independent functioning. Children between the ages of 2 and 18 can be screened for autism using the Social Communication Questionnaire (53). One study found that the Social Communication Questionnaire score of obese girls 6–12 years of age was higher than that of non-obese children, indicating that obese girls have poorer social function. An analysis suggested that this phenomenon may be related to physical self-awareness (54, 55). Social norms have a important effect on the self-perception of children, and discrimination and repellence attributable to obesity may be involved in or even promote the development of DDs.

Notably, the WWI and prevalence of childhood DDs of children 6–11 years of age had a J-shaped relationship, with the turning point set at 11.6. This indicated that children aged 6–11 had the lowest prevalence of DDs when the WWI was 11.6. When the WWI exceeded 11.6, the prevalence of DDs increased significantly with the increasing WWI; this finding is consistent with the main trend observed during this study. A potential link between central adiposity (as indicated by the WWI) and the prevalence of childhood DDs has been previously elucidated. When the WWI was less than 11.6, the prevalence of DDs decreased with the increasing WWI; however, this difference was not significant. A lower WWI may suggest a lower body fat content, indicating potential nutritional deficiencies. Malnutrition is linked to impaired neurodevelopment and cognitive and behavioral issues (56, 57). Additionally, DDs in children have multiple contributing factors. This study considered multiple potential confounding variables; however, other potential factors such as genetics, pollution, nutrition, psychological state, and living environment cannot be excluded. This was a limitation of this study. Therefore, when the WWI is less than 11.6, other dominant factors that affect the incidence of DDs may exist, and the impact of the WWI is relatively small, resulting in a statistically negative relationship between the WWI and prevalence of DDs among children. This phenomenon remains unclear; however, the current evidence indicates that aiming for a low WWI to prevent DDs is insufficient, and that maintaining the WWI near this turning point can reduce the incidence of DDs.

This study had several strengths and limitations. First, we used a national database that can be generalized to a larger group of children by taking into account weighting factors. To confirm the accuracy of the results, it was crucial to use a large sample size to conduct subpopulation analyses. Second, this study proposed a linear positive relationship between the WWI and DDs. Diseases in the NHANES that require children to receive special education or early intervention include ADHD, ASD, learning disabilities, epilepsy, and cerebral palsy. However, this study could not accurately identify any of these diseases. Furthermore, this study is cross-sectional in nature, which means that it cannot establish a causal relationship between WWI and DDs and it does not allow for the dynamic chronological study of the development of WWI in relation to DDs.

In conclusion, children 6–17 years of age with a high WWI were more likely to develop DDs, suggesting that central adiposity and visceral fat may be risk factors for DDs. Additionally, for children 6–19 years of age, the WWI had a threshold effect on the prevalence of DDs. However, further studies should be performed to reveal the underlying mechanism.

## Data availability statement

The raw data supporting the conclusions of this article will be made available by the authors, without undue reservation.

## Ethics statement

The studies involving humans were approved by Research Ethics Review Board of the Center for Health Statistics. The studies were conducted in accordance with the local legislation and institutional requirements. Written informed consent for participation in this study was provided by the participants’ legal guardians/next of kin.

## Author contributions

XL: Conceptualization, Formal analysis, Methodology, Software, Visualization, Writing – original draft. QZ: Funding acquisition, Investigation, Project administration, Supervision, Validation, Writing – review & editing.
